# “Do probiotics mitigate GI-induced inflammation and perceived fatigue in athletes? A systematic review”

**DOI:** 10.1080/15502783.2024.2388085

**Published:** 2024-08-28

**Authors:** R.P. Kearns, J.S.G. Dooley, M. Matthews, A.M. McNeilly

**Affiliations:** Ulster University, School of Life and Health Sciences, Sport and Exercise Sciences Research Institute, Ulster University, Newtownabbey, Antrim, UK

**Keywords:** Exercise, Gut, Probiotic, Microbiome, Fatigue

## Abstract

**Background:**

Fatigue and gastrointestinal (GI) distress are common among athletes with an estimated 30–90% of athletes participating in marathons, triathlons, or similar events experiencing GI complaints. Intense exercise can lead to increased intestinal permeability, potentially allowing members of the gut microbiota to permeate into the bloodstream, resulting in an inflammatory response and cascade of performance-limiting outcomes. Probiotics, through their capacity to regulate the composition of the gut microbiota, may act as an adjunctive therapy by enhancing GI and immune function while mitigating inflammatory responses. This review investigates the effectiveness of probiotic supplementation on fatigue, inflammatory markers, and exercise performance based on randomized controlled trials (RCTs).

**Methods:**

This review follows the Preferred Reporting Items for Systematic Reviews and Meta-Analysis (PRISMA) guidelines and PICOS (Population, Intervention, Comparison, Outcome, Study design) framework. A comprehensive search was conducted in Sportdiscus, PubMed, and Scopus databases, and the screening of titles, abstracts, and full articles was performed based on pre-defined eligibility criteria. Of the 3505 records identified, 1884 were screened using titles and abstracts, of which 450 studies were selected for full-text screening. After final screening, 13 studies met the eligibility criteria and were included for review. The studies contained 513 participants, consisting of 351 males and 115 females, however, two studies failed to mention the sex of the participants. Among the participants, 246 were defined as athletes, while the remaining participants were classified as recreationally active (*n* = 267). All trials were fully described and employed a double- or triple-blind placebo-controlled intervention using either a single probiotic strain or a multi-strain synbiotic (containing both pro- and pre-biotics).

**Results:**

This review assesses the effects of daily probiotic supplementation, ranging from 13 to 90 days, on physical performance and physiological markers in various exercise protocols. Ten studies reported improvements in various parameters, such as, enhanced endurance performance, improved anxiety and stress levels, decreased GI symptoms, and reduced upper respiratory tract infections (URTI). Moreover, despite no improvements in maximal oxygen uptake (VO_2_), several studies demonstrated that probiotic supplementation led to amelioration in lactate, creatine kinase (CK), and ammonia concentrations, suggesting beneficial effects on mitigating exercise-induced muscular stress and damage.

**Conclusion:**

Probiotic supplementation, specifically at a minimum dosage of 15 billion CFUs daily for a duration of at least 28 days, may contribute to the reduction of perceived or actual fatigue.

## 1. Introduction

Endurance sport participation continues to grow globally, with a 49% increase in marathon runners since 2008 [1] and ultra-events growing 57.8% between 1996 and 2018 [2]. Endurance exercise involves the prolonged maintenance of constant or self-regulated power over a given distance [3–6]. To sustain exercise performance, athletes require an integration of multiple physiological and psychological systems working in conjunction to regulate exercise intensity and fatigue management [7,8]. However, during extended physical exertion, the equilibrium of these systems can be disrupted, detrimentally influencing performance due to factors such as oxidative stress [9], compromised intestinal permeability [10], muscle damage [11], systemic inflammation [12] and immune responses [13]. Among these symptoms, gastrointestinal (GI) distress frequently emerges as a consequence, representing a prevalent performance-inhibiting factor with an estimated 30–90% of participants in endurance events experiencing GI complaints [14]. However, due to a paucity in the research, the etiology of these symptoms remains elusive [14–16]. Nonetheless, two physiological theories have been suggested to elucidate the causative factors [17,18]. One theory is *via* the circulatory-gastrointestinal pathway resulting in a redistribution of blood flow to working muscles, reducing oxygen and nutrients to the splanchnic region resulting in splanchnic hypoperfusion, and subsequent ischemia [19–21]. A second theory is by activation of the neuroendocrine-gastrointestinal pathway, with recent evidence suggesting gut permeability can also influence neural outputs *via* the “Gut-Brain-Axis” [22]. Under certain circumstances, such as during systemic inflammation or infection, pro-inflammatory cytokines can upregulate the enzyme indoleamine 2,3-dioxygenase (IDO), which catalyzes the conversion of tryptophan into N-formyl-kynurenine, initiating the kynurenine pathway resulting in the production of neuroactive compounds such as kynurenic acid and quinolinic acid [23]. Kynurenic acid is thought to be a neuroprotective substance, quinolinic acid, conversely, is neurotoxic [24,25]. Furthermore, the kynurenine pathway is also involved in the regulation of tryptophan metabolism [25], a key amino acid involved in the synthesis of serotonin, a neurotransmitter that regulates mood and fatigue. Upregulation of quinolinic acid can, therefore, indirectly downregulate serotonin production and thus, affect neural drive contributing to feelings of sadness and increased perceptions of fatigue, potentially influencing physical performance [26–28].

Probiotics are live micro-organisms which, when consumed in adequate amounts, confer a health benefit to the host [29] Evidence suggests that probiotics may enhance gut and systemic immune function by improving low-grade inflammation [30,31] and promoting mucosal integrity of the endothelial lining [32]. Probiotics may also aid in maintaining the composition of the microbiota, which encompasses a collective of protozoa, archaea, eukaryotes, viruses, and predominantly bacteria that live symbiotically within humans [33–37]. Several studies have shown that probiotics supplementation could improve immune function in fatigued athletes [38,39] and reduce upper respiratory tract illness (URTI) [40], GI symptoms [38,41] and gut permeability [42]. However, it is unclear whether probiotics are effective in mitigating GI-induced inflammation and perceived fatigue in athletes.

This review aims to systematically examine the data from this unique and fast-growing area of research. By assessing and collating RCTs of the highest quality, the findings from multiple studies were analyzed to identify any patterns or relationships between inflammation, probiotic supplementation, and athletic performance.

## 2. Methods

### 2.1. Search strategy

Studies were identified, screened, and analyzed using the Preferred Reporting Items for Systematic Reviews and Meta-Analysis (PRISMA) statement guidelines [43]. Three electronic databases were searched, PubMed, Scopus, and SportDiscus, up to 1 June 2023. The search focused on four main concepts: probiotics, inflammation, fatigue, and exercise. The search incorporated keywords, searched in specific fields (title, abstract, author supplied keywords) and subject headings. A standardized search strategy for key search terms and phrases was combined with Boolean operators to ensure two lists of combination words related to the intervention and outcome of interest, were generated. These included Probiotics/OR Psychobiotics/OR Synbiotics AND Inflammation/OR Inflammatory, Exercise/OR Athlete AND Fatigue/Or Tiredness [44–49].

### 2.2. Screening and data extraction

**Figure 1.**
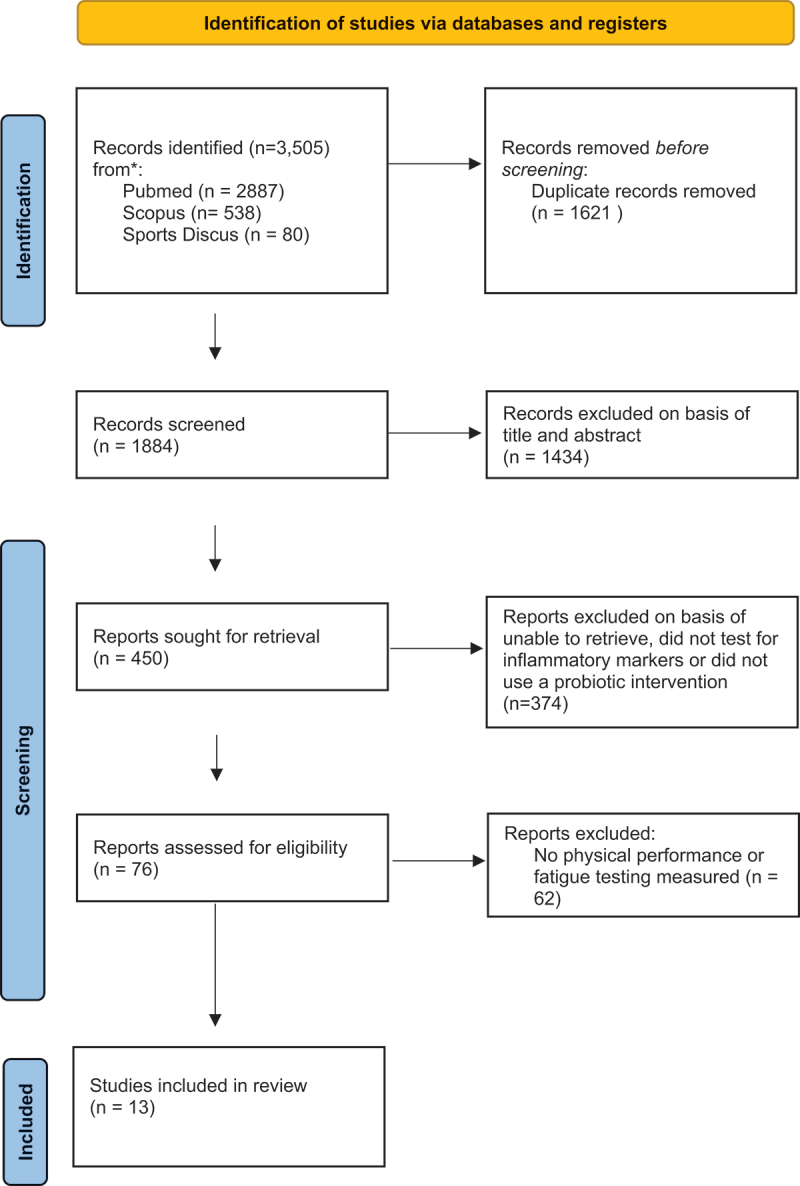
Preferred reporting items for systematic reviews and meta-analysis study flow diagram.

The titles and abstracts from each database were screened by two authors (R.K and A.McN) to determine eligibility. Following the removal of duplicates, a two-phase search strategy was employed. In the initial phase, the eligibility of the research studies was evaluated in accordance with the PICOS criteria (Appendix A) [50,51]. This assessment also involved analysis of subject titles and abstracts, comparing them against an inclusion and exclusion criteria. Studies which had questionable suitability were included with a final decision to keep or remove agreed in phase two. Phase two involved the full articles being retrieved and assessed against an eligibility criterion. Studies were considered eligible if they contained description of participants (athlete or non-athlete), sample size, study design, interventions used (including frequency, dose, strain, and strain designation of probiotic supplementation), and key outcomes of interest (inflammatory biomarkers, performance improvement, and fatigue). Only randomized controlled trials were considered, with the inclusion period spanning from 2012 to 2023 to ensure the inclusion of up-to-date findings and the use of contemporary methods [52,53]. The study population consisted of human participants over the age of 18 years. Risk of bias (ROB) was assessed using the latest version of the Cochrane Collaboration risk of bias tool for randomized controlled trials [54]. Any differences in opinion relating to study eligibility were resolved through discussion. The study selection process is summarized in Figure 1.

## 3. Results

Of the 3505 records identified, 1884 were screened using titles and abstracts, of which 450 studies were refined for full-text screening. After final screening, 13 studies meet the eligibility criteria and were included for review (Figure 1). The studies varied in quality with six RCTs rated as high quality/low rate of bias (ROB) [55–61] and the remaining seven studies were considered acceptable ROB (Table 1).

**Table 1.** Summary of main findings. +, acceptable ROB; ++, high quality/low ROB; -, low quality/high ROB.

Author, year	Human Participants (n)	Probiotic Intervention & Duration	Fatigue Analysis	Performance Analysis	Inflammation Analysis	Primary outcome of Intervention	Risk of Bias (++,+,-)
Komano et al., 2018	*n*= 51 (M)	1x10(10)*LactococcusD2:D10 lactis* JCM 5805 (LC Plasma) − 13 Days	Daily questionnaire, based on perceived fatigue. Significant reduction in URTI symptoms.	No details on training regime. No significant difference.	CPK, LDH, and adrenaline. No significant difference.	Relieves symptoms of URTI and decreases fatigue accumulation during consecutive high intensity exercise in athletes	(+)
Huang; Lee; Lee; et al., 2019.	*n*= 54 (27 M & 27 F)	1 × 10(10) or 3 × 10(10) *Lactiplantibacillus plantarum* TWK10–6 weeks	Reductions in lactate and ammonia levels suggest potential fatigue-reducing. Not Significant.	TWK10 groups significant increase in endurance time	NLR and PLR as markers of inflammation. No significant difference	Significantly improved exercise endurance performance in a dose-dependent manner	(+)
Huang; Wei; Huang; et al., 2019	*n*= 34	3x 10(10) *Lactiplantibacillus plantarum* PS128–4 weeks	LDH, ammonia, lactate, and free fatty acids (FFA) were analysed in the serum.	Wingate test and VO_2_ test.	Inflammatory cytokines (TNF-α, IFN-γ, IL-2, IL-4, IL-6, IL-10), analysed.	Significantly decreased CK content. pro- and anti- inflammation cytokines, and oxidative stress (MPO) markers were Significantly improved	(+)
Huang; Pan; Wei; et al., 2020	*n*= 20 (M)	3x10(10) *Lactiplantibacillus plantarum* PS128–4 weeks	Treadmill exercise protocol. RPE results not mentioned	VO_2_ and body composition. No Significant difference	CBC analysis and biochemical variable analysis. No Significant difference	Improvement in endurance running performance, but not VO_2_	(++)
Strasser et al., 2016	*n*= 29 (13M & 16F)	1x10(10) 6 strains: *Bifidobacterium bifidum* W23, *Bifidobacterium lactis* W51, *Enterococcus faecium* W54, *Lactobacillus acidophilus* W22, *Levilactobacillus brevis* W63, and *Lactococcus lactis* W58–3 months	Fails to provide details about how fatigue was measured and outcomes	VO_2_, No Significant difference	Neopterin levels assessed. No significant difference	Reduced exercise-induced drops in tryptophan levels and reduced the incidence of URTI	(++)
Quero et al., 2021	*n*= 27(M)	1x10(9) 4 strains: *Bifidobacterium lactis* CBP-001010, *Lacticaseibacillus rhamnosus* CNCM I-4036, *Bifidobacterium longum* ES1, and fructooligosaccharides − 30 days	The Brief Fatigue Inventory (BFI) questionnaire. No Significant difference	VO_2_ no significant difference	Cortisol, CRH, ACTH assessed. No SIG difference	Improvement in anxiety, stress, and sleep quality,	(+)
Pugh et al., 2019	*n*= 24 (20M & 4F)	2.5(9) − 4 strains: *Lactobacillus acidophilus* CUL60, *L. acidophilus* CUL21, *Bifidobacterium bifidum* CUL20, and *Bifidobacterium animalis subsp*. *Lactis* CUL34–28 days	Fatigue was not explicitly tested or reported in the methods section.	Marathon, GI symptom severity significant reduction resulting in improved speed	IL-6, IL-8, IL-10, and cortisol. No significant difference	lower incidence and severity of GI symptoms in marathon runners	(+)
Roberts et al., 2016	*n*= 30 (25 M & 5 F)	3x10(10) LAB(4)Anti 0 billion CFU·day−1 *Lactobacillus acidophilus* CUL-60 (NCIMB 30,157), 10 billion CFU·day−1 *Lactobacillus acidophillus* CUL-21 (NCIMB 30,156), 9.5 billion CFU·day−1 *Bifidobacterium bifidum* CUL-20 (NCIMB 30,172) and 0.5 billion CFU·day−1 *Bifidobacterium animalis subspecies lactis* CUL-34 (NCIMB 30,153)/55.8 mg·day−1 fructooligosaccharides/400 mg·day−1 α-lipoic acid, 600 mg·day−1 N-acetyl-carnitine). OR LAB(4) containing same strains but not antioxidants	RPE - Results failed to mention outcome	Long distance triathlon. No significant differences	LAB4ANTI significantly reduced endotoxin units	May reduce endotoxin unit levels	(++)
Salleh et al., 2021	*n*= 30	3x10(10) *Lacticaseibacillus casei* Shirota- 6 weeks	Assessed through anxiety, stress, and mood questionnaires. Significant reduction	Measured through aerobic capacity, hand strength, leg power, speed, and agility tests. Not significant	Inflammation assessed through questionnaire - anxiety and stress. significant difference	Improved aerobic capacity and relieved anxiety and stress	(+)
Schreiber et al. 2021	*n*= 30 (M)	1.5 × 10^9 CFU 5 strains: at least (≥) 4.3 × 10^9 CFU *Lactobacillus helveticus Lafti* L10 (28.6%), ≥4.3 × 10^9 CFU *Bifidobacterium animalis ssp. lactis Lafti* B94 (28.6%), ≥3.9 × 10^9 CFU *Enterococcus faecium* R0026 (25.7%), ≥2.1 × 10^9 CFU *Bifidobacterium longum* R0175 (14.3%) and ≥0.4 × 10^9 CFU *Bacillus subtilis* R0179 (2.8%) − 90 Days	Time-to-fatigue (TTF) test. Significant improvement	(VO_2_) and ventilatory threshold (VTh). No significant difference	CRP IL-6, and TNFα. Not significant.	Reduce the incidence and severity of GI symptoms, and RPE of elite endurance athletes	(++)
Komano et al., 2023	*n*= 37 (M)	1x10(11). *Lactococcus lactis* strain Plasma (LC-Plasma) − 14 Days	low frequency to high frequency ratio (LF/HF). Significant improvement	metabolic rate–hour (MET-h) based on energy expenditure. No significant difference.	(TGF-β, IL-6, cathepsin L, adrenaline, 8-OHdG, testosterone, leptin, and CPK. No significant difference	Autonomic fatigue parameters (LF/HF) were significantly lower in the LC-Plasma group.	(++)
Lee; Ho; Hsu; et al. 2022	*n*= 114 (78 M & 36 F)	1x 10(10) *Lactobacillus paracasei* PS23 live bacteria group (1 × 1010 CFU/capsule, 2 capsules/day) (L-PS23), and a PS23 heat-killed bacteria group (1 × 1010 cells/capsule, 2 capsules/day) (HK-PS23) − 6 weeks	Heat killed bacteria fatigue index was significantly greater in the placebo. Probiotic group not mentioned	CMJ, IMTP, and Wingate Anaerobic Test. Significantly reduced muscle force loss	CK, high-sensitivity CRP, myoglobin, testosterone, and TBARS. Significant improved	Improved performance, reduced fatigue and recovery	(++)

### 3.1. Population and study characteristics

The 13 remaining studies contained 513 participants, consisting of 351 males and 115 females, however, two studies failed to mention the sex of the participants [62,63]. Among the participants, 246 were defined as athletes, with athlete status ranging from duration in sport [57,62] to representation at national level [60], while the remaining participants were classified as recreationally active (*n=*267). All studies utilized at least a double-blind placebo-controlled trial and all trials were fully described using either a single probiotic strain or a multi-strain symbiotic (Table 1). Interventions included taking a daily probiotic for different durations, ranging from 13 days in one study to 12 weeks in another and included various strains, colony-forming units (CFUs), and dosages (Table 1). Seven of the interventions involved supplementation using a single-strain probiotic bacterium: *Lactococcus lactis* JCM 5805 (LC Plasma) [55,56], *Lactiplantibacillus plantarum* TWK10 [61,64] *Lactiplantibacillus plantarum* PS128 [57,62] and *Lacticaseibacillus casei* [63]. One study used the same *Lacticaseibacillus paracasei* PS23 strain but utilized a heat-killed and a live version [65]. Three studies utilized a combination of complementary probiotic strains: *Bifidobacterium bifidum* W23, *Bifidobacterium lactis* W51, *Enterococcus faecium* W54, *Lactobacillus acidophilus* W22, *Levilactobacillus brevis* W63, *and Lactococcus lactis* W58 [58]; *Lactobacillus acidophilus* CUL60, *L. acidophilus* CUL21, *Bifidobacterium bifidum* CUL20, *and Bifidobacterium animalis subsp. Lactis* CUL34 [66]; *Lactobacillus helveticus Lafti* L10, *Bifidobacterium animalis ssp. lactis Lafti* B94, *Enterococcus faecium* R0026, *Bifidobacterium longum* R0175, *Bacillus subtilis* R0179 [60]. The remaining two studies used products containing multiple probiotic strains and synbiotics, which included a combination of *Bifidobacterium lactis* CBP-001010, *Lacticaseibacillus rhamnosus* CNCM I-4036, *Bifidobacterium longum* ES1 and fructooligosaccharides (a type of prebiotic) [67] *Lactobacillus acidophilus* CUL-60 and *Lactobacillus acidophillus* CUL-21, *Bifidobacterium bifidum* CUL-20, *Bifidobacterium animalis subspecies lactis* CUL-34, and fructooligosaccharides (Roberts et al., 2016).

### 3.2. Changes in physical performance and characteristics

A range of modalities were used to assess changes in physical performance, including maximal oxygen uptake (VO_2_) [57,60,62,64,66], incremental exercise tests [58,62,63,65,66], performance in competitive events (triathlons) [59,62], lactate threshold [66], calculating metabolic rate-hour [55,65,67], multi-stage shuttle run tests [63,65] and various strength and power tests [65]. Probiotics displayed mixed results on blood work analyzing lactate, creatine kinase (CK), and ammonia mitigation, with two studies showing no effect [55,62] and one study reporting significant benefits [57]. Eight studies [57,58,60–65] analyzed body composition using a range of methods. Two studies [57,62] used dual-energy X-ray absorptiometry (DEXA), five used bioelectrical impedance analyzers (BIA) [56,58,61,63,64] and one utilized Skinfold Calipers [60]. No studies found significant differences in body composition before and after supplementation with probiotics. Upper respiratory tract infection (URTI) was assessed by two of the studies, with both showing a daily probiotic reduced the incidence of URTI symptoms during the duration of trial [55,58]. Other significant findings included lower autonomic fatigue parameters (LF/HF) [56], reduction in rate of perceived exertion (RPE) [60], improvements in exhaustion time [64], reduction in ammonia production [61,64] and improvements in anaerobic and aerobic exercise [62,63].

### 3.3. Biomarker changes

All 13 studies assessed a range of markers relating to systemic inflammation. CD86 and HLA-DR expression on plasmacytoid dendritic cells (pDCs) are measured as biomarkers of immune activation and maturation, indicating inflammation. Results showed that CD86 expression on pDCs was significantly increased in the LC-Plasma group compared to the placebo [55]. However, there were no significant differences in the HLA-DR expression on pDCs [55]. Two studies investigated neutrophil-to-lymphocyte ratio (NLR) and platelet-to-lymphocyte ratio (PLR), producing mixed results, with one study [64] showing no significant differences between TWK10 and placebo, whereas the second study [61] investigating TWK10 found a significant decrease in NLR and PLR, indicating reduced inflammation. Multiple studies investigated inflammatory biomarkers including IL-6, IL-8, TNF-α, IL-10, and IgG, with mixed results [56,57,59,60,62,67]. *L. plantarum* PS128 supplementation significantly reduced intense exercise-induced inflammation markers such as TNF-α, IL-6, and IL-8 [62]. Another study found no significant changes in IL-6 and CRP values, however, the probiotic group showed lower mean TNF-α values compared to the control group [60]. Synbiotic intervention also showed no significant differences in IL-1β and IL-10 concentrations between groups, and immunoglobulin A levels did not show significant variations [67]; moreover, no significant changes were found in sCD14, LR, I-FABP, IL-6, IL-8, IL-10, cortisol, and ACTH [66]. Regarding endotoxin units (EU) and IgG endotoxin-core antibody levels, the probiotic group (LAB4ANTI) exhibited a significant reduction in EU levels in both pre-race and 6 days post-race [59]. IgG anti-EU concentrations were significantly lower in the LAB4ANTI group compared to the LAB4 and placebo groups at baseline [59]. CK, myoglobin, TBARS, hs-CRP, and testosterone levels were also analyzed as markers of exercise-induced muscle damage and inflammation. The probiotic groups (L-PS23 and HK-PS23) demonstrated significantly lower increases in these markers compared to the placebo group [65]. Finally, one study utilized validated psychological scales to evaluate mental well-being, revealing that the probiotic group demonstrated significant reductions in anxiety and stress levels in comparison to the control group, suggesting potential indirect effects on inflammation markers [63].

### 3.4. Fatigue markers

In terms of fatigue assessment, there was great heterogeneity across the studies. All studies included a fatigue element but used different protocols such as subjective questionnaires [55,56,59,63,67], markers of muscle damage or metabolic by-products [55,58,62,65], improvements in performance from baseline [57,59], and RPE [60,61,64,66]. Four studies assessed RPE and found that probiotic supplementation improved perceived exhaustion time [60,61,64,66]. The studies investigating markers of muscle damage and metabolic by-products such as lactate accumulation, CK, CPK, ammonia, and Tryptophan produced mixed findings [55,56,58,61,62]. One study showed CK was significantly reduced following probiotic supplementation, but other indices related to muscular injury (e.g. LDH, protein carbonyl, myoglobin) and fatigue (lactate, ammonia, FFA) remained unchanged after the triathlon competition [62]. Whereas another study showed that probiotic supplementation significantly reduced lactate and ammonia concentrations [61]. Two studies found no significant difference in CK [56,61]. One study found that probiotics reduced TRP degradation rates [58]. Two studies assessed fatigue based on performance time compared to baseline scores, with both showing improvements following probiotic supplementation [57,59], however, only one study found performance increased significantly [57]. Two studies assessed fatigue symptoms when investigating the occurrence of URTI [55,58], both finding that probiotic supplementation reduced URTI symptoms and, therefore, fatigue by association.

Three studies examined GI complaints such as nausea, cramps, diarrhea, stomach pain or discomfort, urge to vomit or defecate, etc., using subjective questionnaires. Two of these studies found a significant improvement [59,66], and the other showed a non-significant improvement [60]. However, only three of the studies [59,60,66] utilized a form of GI symptom test, one study [66] used a rating scale [68], whereas the other two studies [59,60] based their questionnaires on previously published peer-reviewed journals [69,70]. All three studies recorded significantly lower GI symptoms in the groups taking PRO supplementation compared to the control.

## 4. Discussion

The aim of this study was to examine the data from high-quality RCTs to identify any patterns or relationships between inflammation, probiotic supplementation, and athletic performance.

Analysis of the 13 studies revealed that some interventions induced positive effects in terms of inflammation, fatigue, and GI symptom reduction.

### 4.1. Effect of probiotics on fatigue

It is well known that fatigue development during endurance performance is largely determined by a complex interplay between psychophysiological and physical capacities [71,72]. All studies found a positive correlation between taking probiotic supplementation and a reduction in fatigue, fatigue causing symptoms or perceptions of fatigue. This is in line with existing literature suggesting that probiotics may have positive psychological benefits through interactions with the GBA [73]. However, there is great heterogeneity in the methods used to assess fatigue across the studies. For example, several studies assessed fatigue using questionnaires and scales [58–60,63,64], such as, Borg’s Rate of Perceived Exertion (RPE) [60,64] which is a valid and reliable method for monitoring internal training loads in athletes [74,75]. Other scales included “The Brief Fatigue Inventory” (BFI) [67] and “The Perceived Stress Scale (PSS) questionnaire” [63]. GI symptom scales were also utilized as they included a subjective fatigue element [59,60,66]. Overall, the findings suggest a correlation between decreased GI complaints and reduced perceived fatigue in these studies.

Fatigue was also assessed through biomarker analysis, such as lactate and serum concentrations of TRP and KYN. According to the results, probiotics may offset fatigue by reducing lactate accumulation [64] and TRP degradation [58]. Endurance training increases skeletal muscle mitochondria and type 1 fiber content and fatty acid oxidation, which may explain the lower serum lactate due to higher proportion of energy supplied through fatty acid oxidation instead of carbohydrate [76]. Reduced TRP degradation supports serotonin metabolism and therefore may reduce perceptions of fatigue [77]. Moreover, increased intestinal permeability, psychological stress, reperfusion injury during prolonged exercise, and elevated circulatory pro-inflammatory cytokines may be a result of higher levels of kynurenines [58]. These factors have been associated with negative effects on mood and cognition, which can have implications for athletic performance [78]. Additionally, two studies reported a significant reduction in anxiety and stress [63,67]. These findings are further reinforced by Adikari and colleagues [79] who observed a notable decrease in competitive anxiety and perceived stress among 20 football players following 8 weeks of daily probiotic supplementation.

### 4.2. Effects of probiotics on inflammation

The studies in this investigation tested a range of inflammatory biomarkers, including inflammatory cytokines (IL-2, IL-4, IL-6, IL-10, IL-1β, TNF-α), ROS, kynurenines, cortisol, biomarkers for gut permeability and muscle damage. Probiotic supplementation produced mixed results, and two studies of various strains produced positive results in reducing inflammatory biomarkers [62,65]. However, six studies found no significant changes in pro-inflammatory cytokines following probiotic supplementation [55–58,60,67]. These findings contradict previous studies that have suggested a reduction in pro-inflammatory markers with probiotic intervention [31,80,81]. Several factors may contribute to these contrasting results. For instance, the training regimen and exercise protocol employed were not monitored and, therefore, may not have been sufficient to elicit an inflammatory response [55]. Moreover, many of the included studies involved athletes who typically exhibit a higher tolerance for high-intensity exercise [55,57,58,60,67] and may not experience the same level of inflammatory response as other participants [51].

### 4.3. Effect of probiotics on performance

The 13 studies examined a range of ‘performance’ protocols, and VO_2_ was not significantly affected through the administration of probiotics, which is indicative of the previous research that attempted to augment VO_2_ through nutritional interventions with no success [82,83]. Three included studies that recorded an increase in aerobic capacity, which is thought to be a result of regulation of energy balance and metabolism [57,62,63]. One of these studies found probiotic supplementation improved endurance performance significantly by 130% which the authors suggest may be a result of probiotics ameliorating the onset of central and peripheral fatigue mechanisms. The influence of probiotics on aerobic capacity is consistent with a previous review, which reported an increase in oxygen uptake among swimmers who consumed probiotics [84]. These findings contribute to the existing body of research that demonstrates a positive association between probiotic supplementation and a decrease in time to fatigue. This correlation has been observed in preclinical studies [85–87] as well as clinical studies [64,88,89] involving both athletes and non-athletes.

### 4.4. GI complaints

GI complaints are common in endurance sports [9,90] with various degrees of severity, from mild reflux and nausea to vomiting and bloody diarrhea [16,91]. However, only three of the studies [59,60,66] utilized a form of GI symptom test, with Pugh and colleagues [66] using a rating scale [68], whereas the other two studies [59,60] based their questionnaires on previously published peer-reviewed articles [69,70]. All three studies recorded lower GI symptoms in the groups taking probiotic supplementation compared to the control, two significantly [59,66] and one non-significantly [60]. These three studies all used multi-strain probiotics, with CFUs ranging from 15 to 30 billion per dosage for durations between 28 and 90 days. A previous review [38] investigating the efficacy of probiotic supplementation in reducing GI symptoms in athletes produced similar findings, reporting frequency, and severity of GI symptoms were reduced by approximately one-third in athletes supplementing with a multi-strain *Lactobacillus* or *Bifidobacterium* probiotic.

### 4.5. Limitations

Several limitations should be considered when interpreting these findings. Heterogeneity in exercise protocols and daily training regimens across the studies introduces variability in participants’ training status, potentially reflecting different stages in their training cycles. Certain studies also lacked specific assessments, for example, some studies did not measure biomarkers of gut permeability or specific markers of muscle damage [55,56,62,64,65,67]. These assessments could provide valuable insights into the mechanisms underlying the influence of probiotics. Diet standardization was another limitation. While a few studies set limitations on additional supplements, alternative probiotics, fermented foods, and antibiotics, overall diet standardization was lacking [57,64,66,67]. Moreover, dietary intake during races was not recorded, one study [66] did not monitor the use of carbohydrate and water intake, which has been shown in the previous research to have influenced performance outcome due to variability with gastric emptying [92]. The assessment of fatigue also exhibited heterogeneity across the studies. Different protocols and measures were used, making it challenging to compare and generalize the findings related to fatigue.

The search strategy employed in this systematic review focused solely on three electronic databases (PubMed, Scopus, and SportDiscus), potentially excluding relevant studies from other sources and introducing a selection bias. However, the databases selected are robust and contained all peer reviewed high-quality papers. Moreover, the use of PRISMA guidelines ensured a systematic approach to identification and analysis.

Although the study population consisted of a total of 513 participants, there was an imbalance in the distribution between male (351) and female (115) participants. Additionally, two studies did not report the gender of the participants, which limits the generalizability of the findings between males and females. However, the inclusion of a diverse participant population, including both athletes and recreationally active individuals, broadens the applicability of the study findings within these specific populations.

Finally, the probiotic strain specificity and dose dependency of probiotics’ effects were not fully elucidated in our analysis. A nuanced classification of probiotics, based on their established or proposed impacts on inflammation, oxidative stress, or gut health, might elucidate their prospective advantages in enhancing exercise and athletic performance. Additionally, one study [60] indicated *at least (≥) 4.3 × 10^9 CFU* for various strains, introducing ambiguity in the precise quantity of CFUs provided. This range represents a limitation in the clarity and reproducibility of the probiotic dosages administered, potentially influencing the reviews outcomes and interpretations.

## 5. Conclusion

This comprehensive review highlights the potential beneficial effects of probiotic supplementation on blood biomarkers, physical performance, and fatigue. The findings suggest that probiotics, specifically a multi-strained probiotic at a minimum dosage of 15 billion CFUs daily for a duration of at least 28 days, may contribute to the reduction of perceived or actual fatigue. These findings also align with existing literature suggesting that probiotics may exert psychological benefits through their interactions with the gut-brain axis. Notwithstanding, it is important to acknowledge the limitations present in the selected studies, including disparities in probiotic strains, timing, dosage, duration, and testing protocols, as well as the lack of standardized training regimes. Future research should aim to address these limitations, establish standardized protocols, and explore the mechanisms underlying probiotic effects to optimise their utilisation for enhancing exercise performance.

## Appendix A Appendix PICOS criteria (Population, Intervention, Comparison, Outcome, and Study Design)[50,50,]

**Table:**

PICOS	Inclusion Criteria
*Population*	Did the study include human participants? Did the subject include adults (>18 yrs)? Did the study include healthy subjects?
*Intervention*	Type, time, and frequency must be described.
*Comparators*	Controlling of bias is adequate, e.g. double-blind, randomization, etc.
*Outcome*	Did the study state improvements/non-improvements in exercise performance? Did the study state improvements/non-improvements in inflammation biomarkers? Did the study measure fatigue
*Study Design*	Was the study published fully? Was the study primary research?
